# Fetal Central Nervous System Derived Extracellular Vesicles: Potential for Non-invasive Tracking of Viral Mediated Fetal Brain Injury

**DOI:** 10.3389/fviro.2021.782863

**Published:** 2021-11-18

**Authors:** Laura Goetzl, Angela J. Stephens, Yechiel Schlesinger, Nune Darbinian, Nana Merabova, Miriam Hillel, Alec J. Hirsch, Daniel N. Streblow, Antonio E. Frias, Victoria H. J. Roberts, Nicole N. Haese, Arunmani Mani, Yifat Eldar-Yedidia

**Affiliations:** 1 Department of Obstetrics, Gynecology and Reproductive Sciences, McGovern Medical School, University of Texas Health Science Center at Houston, Houston, TX, United States,; 2 Shaare Zedek Medical Center, Jerusalem, Israel,; 3 Center for Neural Repair and Rehabilitation, Shriners Hospitals Pediatric Research Center, Lewis Katz School of Medicine at Temple University, Philadelphia, PA, United States,; 4 Department of Family Medicine, Medical College of Wisconsin-Prevea Health, Green Bay, WI, United States,; 5 The Vaccine and Gene Institute, Oregon Health and Science University, Beaverton, OR, United States,; 6 Division of Pathobiology and Immunology, Oregon National Primate Research Center, Beaverton, OR, United States,; 7 Department of Obstetrics and Gynecology, Oregon Health and Science University, Portland, OR, United States,; 8 Division of Reproductive and Developmental Sciences, Oregon National Primate Research Center, Beaverton, OR, United States

**Keywords:** exosomes/extracellular vesicles (EVs/ECVs), cytomegalovirus (CMV), Zika (ZIKV), Contactin-2, prenatal diagnosis, microcephaly

## Abstract

**Introduction:**

Extracellular vesicles derived from the fetal central nervous system (FCNSEs) can be purified from maternal serum or plasma using the protein Contactin-2/TAG1that is expressed almost exclusively by developing neurons in the hippocampus, cerebral cortex and cerebellum. We hypothesized that fetal CNSEs could be used to non-invasively detect and quantify viral mediated *in-utero* brain injury in the first trimester.

**Materials and Methods:**

First trimester maternal samples were collected from a human clinical population infected with primary cytomegalovirus (CMV) and a non-human primate model of Zika (ZIKV) infection. In the CMV cohort, a nested case control study was performed comparing pregnancies with and without fetal infection. Cases of fetal infection were further subdivided into those with and without adverse neurologic outcome. ZIKV samples were collected serially following maternal inoculation or saline. All ZIKV cases had histopathologic findings on necropsy. Serum was precipitated with ExoQuick solution and FCEs were isolated with biotinylated anti-Contactin-2/TAG1 antibody-streptavidin matrix immunoabsorption. FCE Synaptopodin (SYNPO) and Neurogranin (NG) protein levels were measured using standard ELISA kits and normalized to the exosome marker CD81.

**Results:**

Fetal CNSE SYNPO and NG were significantly reduced in cases of first trimester fetal CMV infection compared to those with infection limited to the mother but could not discriminate between fetal infection with and without adverse neurologic outcome. Following ZIKV inoculation, fetal CNSE SYNPO was reduced by 48 h and significantly reduced by day 4.

**Discussion:**

These data are the first to suggest that first trimester non-invasive diagnosis of fetal viral infection is possible. Fetal CNSEs have the potential to augment clinical and pre-clinical studies of perinatal viral infection. Serial sampling may be needed to discriminate between fetuses that are responding to treatment and/or recovering due to innate defenses and those that have ongoing neuronal injury. If confirmed, this technology may advance the paradigm of first trimester prenatal diagnosis and change the calculus for the cost benefit of CMV surveillance programs in pregnancy.

## INTRODUCTION

Two percent (95% CI 2.1–2.4%) of seronegative women will acquire primary cytomegalovirus (CMV) infection during their pregnancy (1). At birth, 10–15% of congenitally infected neonates will have symptoms; of those 6% will die and up to 90% will develop sequelae such as sensorineural hearing loss (SNHL), developmental delay, cognitive impairment, neuromuscular dysfunction (cerebral palsy), epilepsy, impaired vision function, and possibly autism spectrum disorder. Asymptomatic infants remain at significant risk of developing SNHL before 2 years of age. Congenital CMV infection is responsible for more long-term sequelae than either Down syndrome or Fetal Alcohol Spectrum Disorder (2). Zika virus (ZIKV) is a mosquito borne flavivirus that has also been causally implicated in brain injury and microcephaly following *in-utero* infection (3). The estimated risk of clinically detected central nervous system (CNS) injury following first trimester ZIKV infection ranges from 1 to 13% (4). In an epidemiologic study based on Brazilian data, the peak risk of microcephaly appears to correlate with ZIKV infection between gestational week 14 and 17 (5), but findings of ventriculomegaly and cerebral calcifications have been seen with ZIKV infections as late as the 3rd trimester (6).

Significant barriers exist to the effective and timely diagnosis and treatment of fetal brain injury secondary to *in-utero* viral infections such as CMV and ZIKV. Many maternal viral infections are asymptomatic; therefore, detection of infection would require surveillance programs. However, enthusiasm for costly surveillance programs has been dampened by a combination of challenges in the accurate diagnosis of fetal brain injury, lack of effective *in-utero* interventions such as hyperimmune globulin (7), and limited means to monitor treatment effects in real time. In fetuses destined to develop microcephaly, head circumference measured on prenatal ultrasound may be normal prior to 22 weeks gestation age (weeks GA) and fetal MRI is of limited utility in diagnosing other intracranial abnormalities prior to 18 weeks GA resulting in late diagnosis in the absence of early surveillance programs (8, 9). Finally, definitive fetal diagnosis requires amniocentesis with amniotic fluid viral PCR. Although emerging data suggests that earlier amniocentesis may be accurate, the traditional recommendation is for amniocentesis to be performed 6 weeks from maternal exposure and at >21 weeks’ gestation for CMV (10). This calculus may be shifting as data emerges regarding the efficacy of other early anti-viral therapies. A recent small randomized, double-blind, placebo-controlled trial demonstrated 71% reduction in the fetal infection rate with high dose oral valcyclovir in women with a primary CMV infection in the first trimester (3–12 weeks gestation) (11). Valganciclovir treatment was also associated with resolution of hydrops fetalis and minimal sequelae in a recent case report (12). The missing element to any first trimester strategy for the diagnosis and treatment of *in utero* viral infections is a non-invasive first trimester assay similar to cell-free DNA based tests for aneuploidy. An ideal first trimester test would be able to detect fetal injury, especially neurologic injury, prior to seroconversion and have improved sensitivity. Serial testing would ideally be available to determine if treatment was effective and to distinguish between those fetuses at low and high risk for adverse neurologic outcomes prior to mid-trimester ultrasound findings.

Exosomes/extracellular vesicles (ECVs) are membrane-bound endosome-derived nanovesicles that allow the intercellular transfer of genetic material, cytoplasmic and membrane proteins, lipids, miRNAs and other intracellular materials (13). Exosomes freely cross the blood-brain barrier and the placenta through unknown mechanisms, carrying proteins and micro RNAs (miRs) into the peripheral blood while protecting them from degradation (14, 15). We have published a series of foundational investigations demonstrating novel techniques to isolate ECVS derived from the fetal CNS from maternal serum using the protein Contactin-2/TAG1 (CTN-2/Tag-1) that is expressed almost exclusively by developing neurons in the hippocampus, cerebral cortex and cerebellum (16–18)^1^. A small proportion of CTN-2/TAG-1 ECVs may arise from tissues such as His-Purkinje cells (19) but the relatively small contribution of these tissues is unlikely to hinder assay performance. Based on comparisons of concentrations between pregnant and non-pregnant subjects, we have demonstrated that 97% of CTN-2/TAG-1+ ECVs isolated from maternal serum are fetal in origin (16); the 3% contamination by maternal neural ECVs reflects low levels of ongoing neural plasticity in adults. We have demonstrated that the placenta is not a source of CTN-2/TAG-1 (17). Taken together, our work demonstrates that CTN-2/TAG-1 can be used to isolate fetal CNS derived ECV (CNSEs) from maternal blood to generate novel biomarkers. We hypothesized that protein markers of neurologic injury quantified in CTN-2/TAG-1+ ECVs purified from maternal blood could identify fetal CNS injury secondary to *in-utero* viral infection with CMV or ZIKV.

## MATERIALS AND METHODS

### Clinical Recruitment CMV

Clinical recruitment was approved by the local ethics committee of Shaare-Zedek Medical Center and written informed consent was obtained from each participating woman. The study was performed according to Good Clinical Practice (GCP) guidelines.

Samples were collected from pregnant women who were diagnosed with primary CMV infection as previously described (20, 21). Briefly, the diagnosis was made by one of the following serological findings: CMV-specific IgG seroconversion or the presence of low avidity IgG antibodies or CMV-specific IgM with no previous IgG antibodies. The timing of primary infection was determined by the time of seroconversion and/or analysis of the increment of IgG avidity and/or by clinical symptoms. Intrauterine CMV transmission was determined by detection of viral DNA by real-time PCR, either in amniotic fluid or in the newborn’s urine. The analysis of these specimens was performed by the treating physicians in their respective medical centers throughout Israel. Maternal samples collected at the time of diagnosis of primary infection were transferred for biomarker analysis under a uniform transfer of biologic material agreement and with the appropriate permit from the Centers for Disease Control.

### Clinical Outcomes CMV

A nested case control study was performed among women who were infected with CMV and had both stored maternal serum samples and known pregnancy outcome.

Three outcome groups were defined. Non-Transmitters (NT) were defined by the absence of CMV detected in amniotic fluid or fetal urine at birth and by normal neurologic outcome. Asymptomatic Transmitters (AST) were defined by CMV detected in amniotic fluid and/or in fetal urine at birth in combination with the absence of adverse neurologic outcome. Symptomatic Transmitters (ST) were defined by CMV detected in amniotic fluid and/or in fetal urine at birth and abnormal findings in at least one of the following: brain ultrasound, retinal examination, or auditory brain-stem response.

### Non-human Primate ZIKV Model

**FIGURE 1 |:**
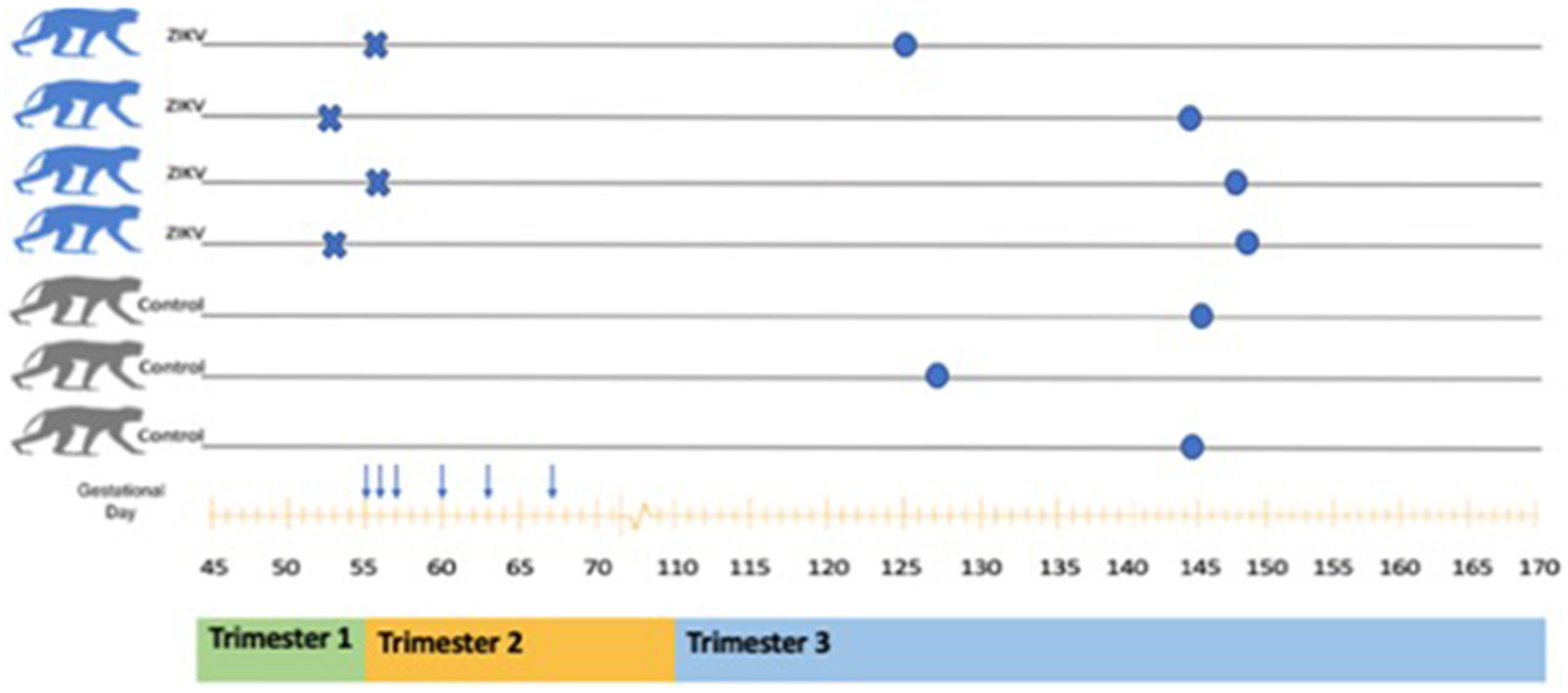
Schematic of animals included in ZIKV analysis. A total of seven pregnant Rhesus Macaques were included (four ZIKV and three Controls). Inoculation with ZIKV_PRVABC59_ is indicated by the symbol X. Serial dam blood sampling is indicated by the symbol ↓. Fetal delivery by cesarean is indicated by the symbol •. A rough correlation with human pregnancy trimesters is shown below the x axis (gestational age in days).

All Zika virus infection experiments utilizing non-human primates were performed in compliance with guidelines established by the Animal Welfare Act for laboratory animal housing and care and in accordance with Oregon National Primate Research Center (ONPRC) Institutional Animal Care and Use Committee approved animal protocol (IACUC #1099). NHP studies were performed in ABSL-2 containment facilities at the ONPRC. Samples used in this study were collected from animals that have been described in detail previously (22). Briefly, on GD 53–55, time-mated pregnant macaques were subcutaneously inoculated with 10^5^ focus forming units (ffu) of ZIKVPRVABC59 as previously described (ZD) or 1 mL of normal saline (CTL) (22, 23). Serial dam serum sample were collected post-inoculation via peripheral blood draws under ketamine sedation, and were sufficient for analysis on days 2–4, 7, 10, and 14. A schematic of the study design is shown (Figure 1). All ZD offspring had histopathologic findings consistent with ZIKV on necropsy.

### Zika Virus Preparation

ZIKV_PRVABC59_ was obtained from the CDC and passaged twice in C6/36 cells [American Type Culture Collection (ATCC)] as previously described (24, 25). Supernatant from infected C6/36 tissue culture was concentrated through a 20% sorbitol cushion and titrated in Vero cells (ATCC) through a focus-formation assay. The viral inoculum was sequenced as previously described (24, 25).

### ECV Isolation and Protein Quantification

Fetal CNS ECVs were purified using previously published techniques (16–18, 26). Briefly, each ~75–125 uL serum aliquot was incubated with thromboplastin-D and a cocktail of protease and phosphatase inhibitors. Supernatants were incubated with exosome precipitation solution (EXOQ; System Biosciences, Inc, Mountainview, California, USA). To isolate the subset of ECVs from neural sources, total ECV suspensions were incubated with monoclonal IgG1 anti-human Contactin-2/TAG1 antibody (clone 372913, R&D Systems, Inc, Minneapolis, Minnesota, USA) that had been biotinylated (EZ-Link sulfo-NHS-biotin System, Thermo Scientific, Inc), and antibody-bound ECVs were precipitated with Streptavidin-Plus UltraLink Resin (Pierce-Thermo Scientific, Inc). Contactin-2/TAG1 is a glycosyl-phosphatidylinositol anchored neuronal membrane adhesion protein of the immunoglobulin superfamily that is transiently expressed in human brain development to guide axonal connections and, in association with other proteins, promote molecular organization of myelinated nerves (27, 28).

The tetra spanning exosome marker human CD81 (American Research Products, Waltham, Massachusetts, USA: Cusabio), and neural markers Synaptopodin (SYNPO) and Neurogranin (NG) were quantified using standard ELISA methods (Reddot Biotech Inc, Kelowna, British Columbia, Canada). The mean value for all determinations of CD81 in each assay group was set at 1.0, and this value was used to normalize their recovery in individual samples.

### Data Analysis

Data analyses were performed using SPSS (IBM SPSS Statistics for Windows, Version 26.0. Armonk, NY). Student’s *t*-testing or ANOVA testing was used to compare means. Correlations were assessed using Spearman’s rank correlation. The *p*-value for significance was set at 0.05.

## RESULTS

### Clinical Nested Case Control CMV Study

**FIGURE 2 |:**
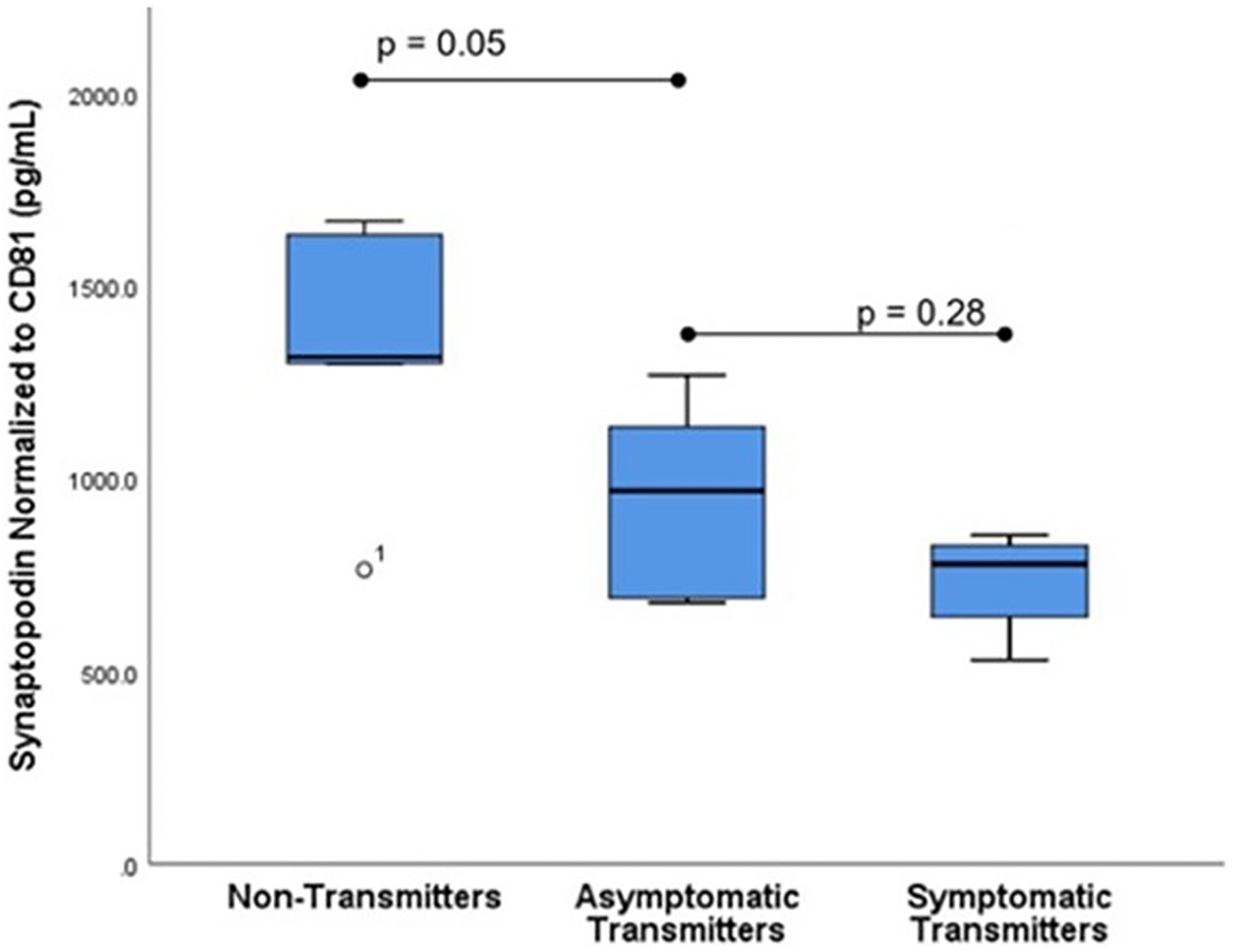
Fetal CNS ECV synaptopodin levels by clinical outcome. ECV protein levels of synaptopodin normalized to CD-81 were compared in three groups: Non-transmitters (NT, *n* = 5), asymptomatic transmitters (AST, *n* = 5), and symptomatic transmitters (ST, *n* = 4). ANOVA testing was used to compare means. The *p*-value for significance was set at 0.05.

**FIGURE 3 |:**
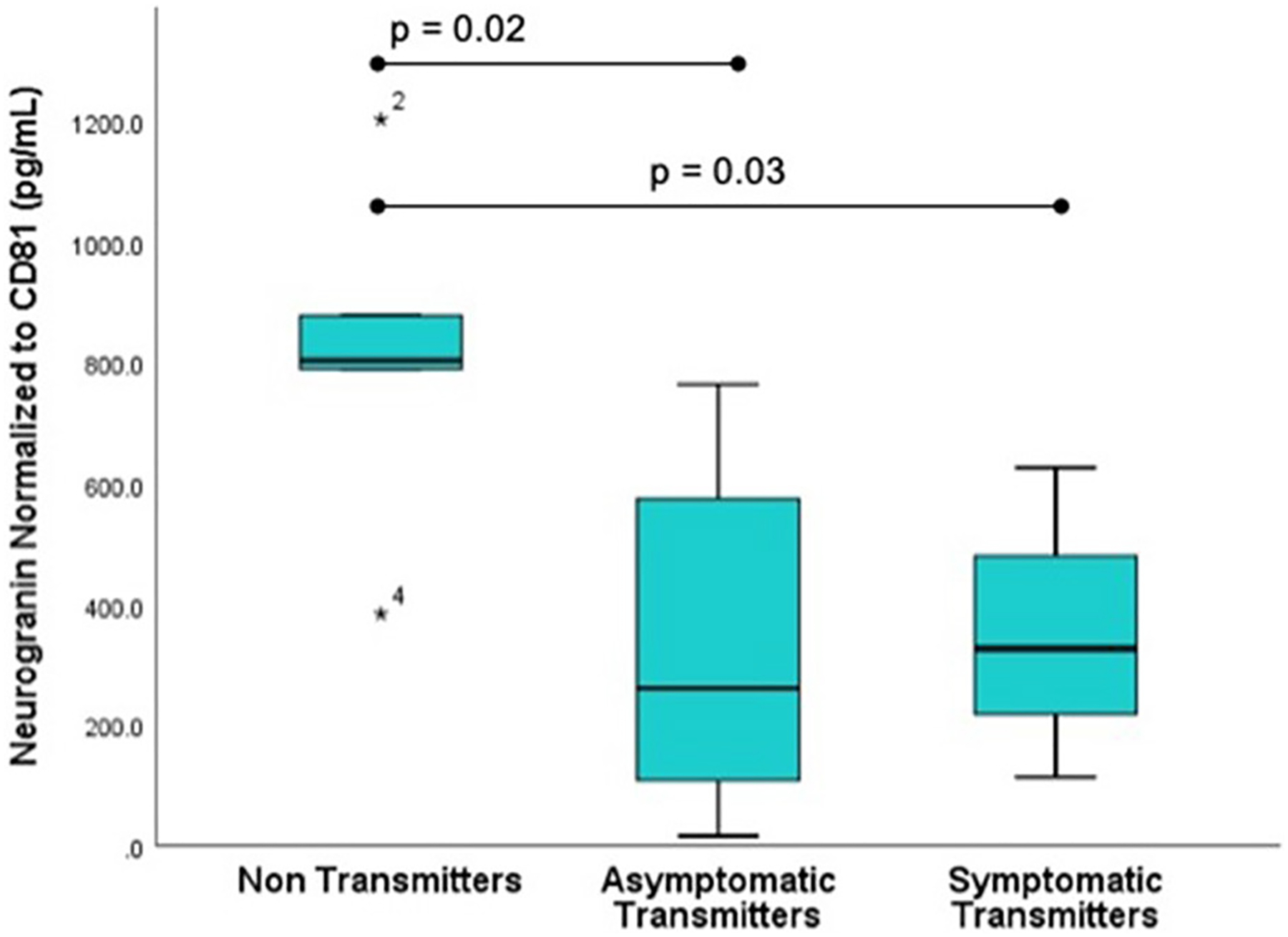
Fetal CNS ECV neurogranin levels by clinical outcome. ECV protein levels of neurogranin normalized to CD-81 were compared in three groups: Non-transmitters (NT, *n* = 5), asymptomatic transmitters (AST, *n* = 5), and symptomatic transmitters (ST, *n* = 4). ANOVA testing was used to compare means. The *p*-value for significance was set at 0.05. * value outside the interquartile range.

Subjects selected from within the original cohort included five non-transmitters (NT), five asymptomatic transmitters (AST), and four symptomatic transmitters (ST). The clinical characteristics of the study subjects are shown (Table 1). For subjects overall the mean maternal age (26.9 ± 3.7 years), mean gestational age (GA) at blood sampling (13.1 ± 3.1 weeks), mean GA at CMV infection (6.8 ± 1.7 weeks), or interval between infection and blood collection (6.3 ± 2.8 weeks) was not different between the three clinical groups (*p* > 0.05). Mean ECV SYNPO levels were significantly reduced in both groups with fetal infection (AST and ST) compared to pregnancies without fetal infection (NT; *p* = 0.04, Figure 2). While ECV SYNPO levels were lower in symptomatic *vs*. asymptomatic fetal CMV infections, this difference was not statistically significant (*p* = 0.28). While the lack of statistical significance might have been due to small samples size, it is clear that there is significant overlap between the two groups and that therefore, it is unlikely that their accurate discrimination/clinical classification at the time of the initial diagnosis of infection. Patient 1 had normal neurodevelopment at age 8 years. This was one of the oldest specimens in the cohort and may have been subject to degradation. Similar results were observed for NG (Figure 3). Of interest, 1 of 4 cases of ST was limited to severe isolated unilateral hearing loss without prenatal findings. Fetal CNSE SYNPO and NG were only partially correlated (*r* = 0.56, *p* = 0.039) suggesting a possible role for multiple markers to improved diagnostic accuracy.

Viral products could not be detected within fetal CNSEs (data not shown).

### Non-human Primate Model ZIKV

**FIGURE 4 |:**
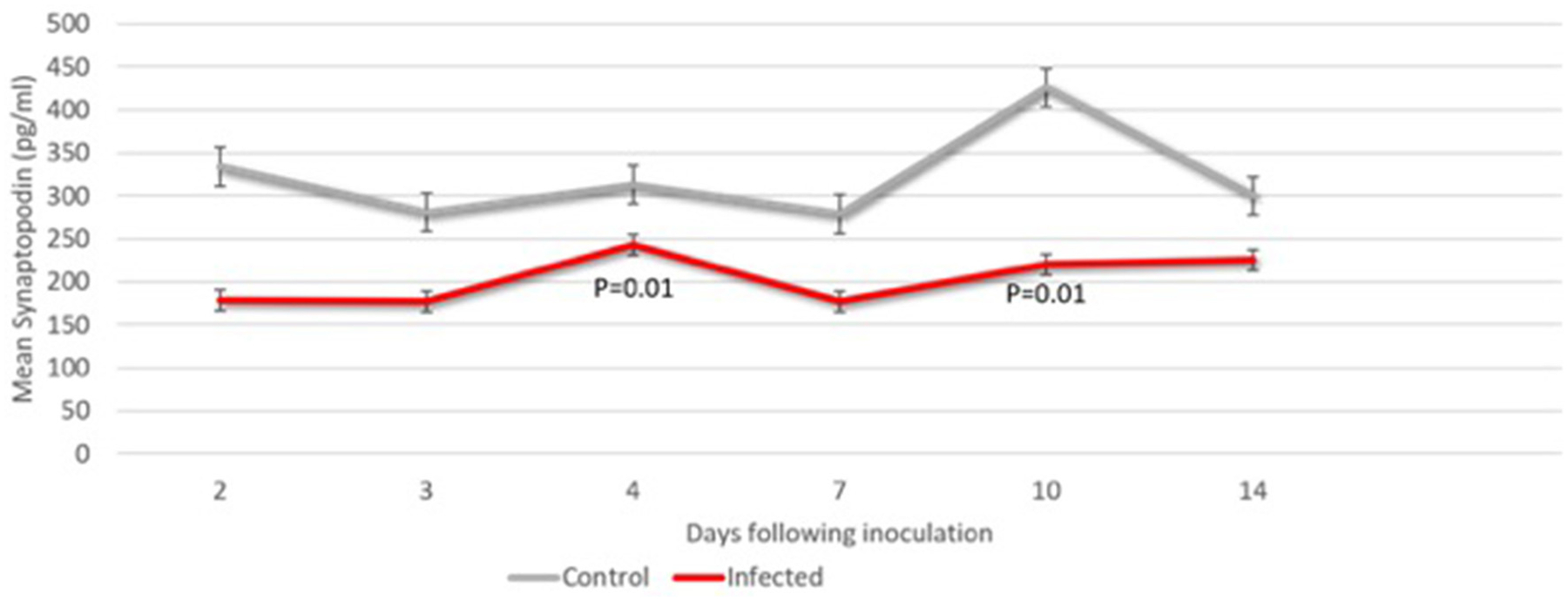
Fetal CNS ECV synaptopodin levels days 2 through 14 following ZIKV vs. saline inoculation. ECV protein levels of synaptopodin normalized to CD-81 were compared in at each timepoint (*n* = 2–4 samples). The Students *t*-test was used to compare means at each time point. The *p*-value for significance was set at 0.05.

Seven fetuses were evaluated for neurologic outcomes (three CTL and four ZD). Decreased levels of fetal CNSE SYNPO in ZIKV infected pregnancies was first observed on Day 2 (Figure 4; Table 2) but did not reach statistical significance until Day 4. Viral RNA could not be detected within fetal CNSEs (data not shown).

## DISCUSSION

These exploratory data suggest that human fetal transmission of CMV can be detected non-invasively using fetal CNSEs isolated from maternal blood as early as 11 weeks gestation and that fetal CNS injury can be detected in a non-human primate model within 2 days of infection. Fetal CNSEs were useful at gestational day 53–55 in the non-human primate model described, which is roughly equivalent to the end of the first trimester in humans. Despite the sample size limitations, these data are the first to suggest that first trimester non-invasive diagnosis of fetal viral infection is possible. If confirmed, this finding could transform the paradigm of prenatal diagnosis for fetal viral infection. Our findings that human Contactin-2 Tag-1 antibody could be used to purify fetal CNSEs in a non-human primate model suggests that this methodology could be used to augment studies of perinatal viral infection in appropriate animal models. Of note, our samples likely represent a mixture of exosomes, other extracellular vesicles and some organelles and we did not attempt to confirm purity due to small sample volumes. However, as opposed to studies where purity is critical (mechanistic and functional exosome studies), biomarkers studies are judged on their ultimate test performance in discriminating between disease and non-disease states and purity is less critical. In future studies, if larger serum/plasma volumes are available, we plan to perform flow cytometry studies to investigate whether or not viral infection alters the proportion of exosomes that care Contactin-2 Tag-1 and/or CD-81.

One surprising finding from our human CMV study was that all fetuses with evidence of *in-utero* infection appear to have significantly reduced levels of injury markers. We hypothesize that all fetuses infected with CMV take an initial “hit” to the CNS but that some are able to rebound secondary to neuroplasticity, and that some are not and are born with neurologic sequelae. Therefore, the most important value of the early samples may be to discriminate between pregnancies with and without fetal transmission to determine which fetuses would benefit from potential interventions but would not provide sufficient information regarding prognosis. Serial sampling may be needed to discriminate between fetuses that are responding to treatment and/or recovering due to innate defenses and those that have ongoing neuronal injury. An addition benefit of fetal CNSE-based prenatal diagnosis is that both initial and serial sampling will be more acceptable to patients since amniocentesis is not necessary. Non-invasive testing would also negate the unavoidable delays in diagnosis using amniocentesis—where the highest sensitivity and specificity are reached after 21 weeks’ gestation. At this gestational age, some CNS injury may be irreversible. If the positive and negative predictive value of CNSE-based prenatal diagnosis is confirmed, it may shift the cost-benefit analysis for CMV surveillance in pregnancy. A recent cost-benefit study concluded that surveillance might be cost effective if anti-viral interventions were more than 30% effective (29), however more precise targeting of those pregnancies that require intervention and serial evaluation would reduce excess costs of treatment and testing from unnecessary treatment.

In addition to markers of neuronal injury, CNSEs may carry viral products or markers of CMV infection that reflects viral specific mechanisms. The similarities between the biogenesis of extracellular vesicles and the release of viral particles from infected cells are intriguing as both processes involved the ESCRT pathway (30). The CMV virus utilizes EVs to enhance viral spread, therefore it is not surprising that EVs can contain viral products (31). CMV specific viral products include the viral envelope proteins gB and gH as well as CMV generated miRs (32, 33). We have attempted to detect gB in CNSEs in our prior CMV studies and have found the results to be inconsistent. A recent study has demonstrated that only 15% of EVs released from CMV infected cells were positive for gB, 5.3% were positive for gH and 3.74% were positive for both gB and gH. The factors that determine the proportion of viral protein positive EVs are not known. Therefore, viral proteins appear to be imperfect biomarkers of CMV infection (34). We were unable to isolate viral genetic products from fetal CNSEs, which was disappointing; we had been hopeful that CNS “viral load” could be estimated non-invasively.

Although viral activity may not be able to be measured directly in fetal CNSEs—there are other potential markers that may reflect perinatal outcomes. EVs from infected cells are known to contain soluble DC-SIGN, a C-type lectin family molecule that increases the susceptibility of recipient cells to CMV but DC-SIGN is found largely in dendritic cells rather than neurons (35). Another study examined total EVs isolated from neonates with active CMV infection. EV levels of the CMV miRs US25-1-5p and UL112-3p were significantly higher in the setting of active CMV infection and were significantly associated with the degree of liver damage secondary to viral hepatitis as assessed by neonatal liver function tests (36). In addition, when free plasma levels of miRs from 13 infants with congenital CMV were compared to controls, levels of miR 183–5p and miR-210–3p were significantly higher (37). This suggests that CNSE miR levels may also be potentially useful in assessing degree of neurologic injury. In addition to the candidates above, there are several other miRs and proteins that have some premise from the literature. Human miR-21 is known to inhibit CMV viral gene expression by targeting Cdc25a, a cell cycle regulator, in neural progenitor cells and may act as an innate anti-viral defense (38). BclAF1 restriction factor, a cellular anti-viral protein, is down regulated by CMV miR-UL112–1 and is also found in ECVs released from human brain cells (39, 40). CMV miR UL112–1 down regulates the antiviral protein Interleukin-32 (IL-32), aiding in evasion of the host immune response (41). In turn, IL-32 is an important regulator of TNF-α, which may adversely affect microglial-mediated neuroinflammation and potentiate fetal brain injury. While TNF-α has not widely been reported in exosomes, tumor necrosis factor-α 1 receptors (TNFR1) levels have been used as a biomarker of response to inhibitors of neuroinflammation in neuronal ECVs (42). Human miR-221 in neural precursor cells inhibits CMV replication by promoting type I interferon and upregulating the activation of NF-kB (43).

In summary, fetal CNSEs appear to have potential in the early identification of viral mediated fetal brain injury. Fetal CNSEs may be useful as early as 11 weeks gestation—and therefore may identify brain injury prior to irreversible damage. Fetal CNSEs offer an advantage as testing can be performed non-invasively, avoiding both the risk and the delay in diagnosis associated with amniocentesis. Fetal CNSEs may also be a useful adjunct to animal models of perinatal viral infection—where the timing of infection is more precisely known. However, larger studies are required to confirm the potential of fetal CNSEs both for endemic viral infections such as CMV and for episodic pandemic infections such as ZIKV and unknown future viral strains.

## Figures and Tables

**TABLE 1 |:** Clinical characteristics of nested case control subjects.

Group	Maternal age	GA at CMV INFX (Weeks)	GA at sampling (Weeks)	Interval	Maternal symptoms	Fetal/neonatal findings
NT	25	8	12	4	Unknown	
NT	26	6	12	6	Unknown	
NT	21	10	16	6	Fever	
NT	26	6.5	10	3.5	Unknown	
NT	27	4	11	7	Fever	
AST	24	6.0	15	9	Unknown	
AST	33	10.5	15	4.5	Fever	
AST	28	5.5	11	5.5	Unknown	
AST	32	6	12	6	Fever	
AST	22	7.5	22	14.5	Unknown	
ST	32	6.0	12	6	Unknown	Microcephaly, intra-cranial calcifications TOP
ST	28	7	11	4	Unknown	Isolated severe unilateral hearing loss
ST	24	8	13	5	Fever	Brain Calcifications, normal development, normal hearing.
ST	28	4	11	7	Unknown	Severe brain damage–TOP

**TABLE 2 |:** Mean fetal neural ECV synaptopodin by day following ZIKV inoculation (ZD) vs. saline injection (CTL).

Day	ZD/CTL (number per group)	SYNPO in ZIKV inoculation group (pg/ml ± SE)	SYNPO in saline inoculation group (pg/ml ± SE)	*p*-value
2	2/3	179.1 ± 39.3	334.4 ± 77.6	0.23
3	4/3	177.3 ± 14.2	281.3 ± 53.7	0.19
4	4/2	243.1 ± 13.6	313.3 ± 0.7	0.01
7	4/2	177.9 ± 23.4	279.3 ± 37.9	0.08
10	4/3	220.3 ± 31.2	426.0 ± 43.9	0.01
14	4/3	226.6 ± 19.1	300.5 ± 80.0	0.45

## Data Availability

The raw data supporting the conclusions of this article will be made available by the authors, without undue reservation.

## Footnotes

1 https://www.proteinatlas.org/ENSG00000184144-CNTN2/tissue.
